# Designing optimal cell factories: integer programming couples elementary mode analysis with regulation

**DOI:** 10.1186/1752-0509-6-103

**Published:** 2012-08-16

**Authors:** Christian Jungreuthmayer, Jürgen Zanghellini

**Affiliations:** 1 Austrian Centre of Industrial Biotechnology, Vienna, Austria; 2 Department of Biotechnology, University of Natural Resources and Life Sciences, Vienna, Austria

**Keywords:** Metabolic engineering, Elementary modes, Minimal cut sets, Integer programming, Strain optimization, Ethanol production, Minimal functionality, Gene regulation

## Abstract

**Background:**

Elementary mode (EM) analysis is ideally suited for metabolic engineering as it allows for an unbiased decomposition of metabolic networks in biologically meaningful pathways. Recently, constrained minimal cut sets (cMCS) have been introduced to derive optimal design strategies for strain improvement by using the full potential of EM analysis. However, this approach does not allow for the inclusion of regulatory information.

**Results:**

Here we present an alternative, novel and simple method for the prediction of cMCS, which allows to account for boolean transcriptional regulation. We use binary linear programming and show that the design of a regulated, optimal metabolic network of minimal functionality can be formulated as a standard optimization problem, where EM and regulation show up as constraints. We validated our tool by optimizing ethanol production in *E. coli*. Our study showed that up to 70% of the predicted cMCS contained non-enzymatic, non-annotated reactions, which are difficult to engineer. These cMCS are automatically excluded by our approach utilizing simple weight functions. Finally, due to efficient preprocessing, the binary program remains computationally feasible.

**Conclusions:**

We used integer programming to predict efficient deletion strategies to metabolically engineer a production organism. Our formulation utilizes the full potential of cMCS but adds additional flexibility to the design process. In particular our method allows to integrate regulatory information into the metabolic design process and explicitly favors experimentally feasible deletions. Our method remains manageable even if millions or potentially billions of EM enter the analysis. We demonstrated that our approach is able to correctly predict the most efficient designs for ethanol production in *E. coli*.

## Background

Arguably the most successful methods in computer aided strain design are based on constraint-based modeling [1]. These methods allow to predict phenotypes by calculating steady state flux distributions through a metabolic network (typically using some kind of flux balance analysis [2]). Various algorithms allow searching for combinatorial gene deletion strategies to optimize the production efficiency of strains [3-6]. These methods utilize an optimization principle, which has been shown to give accurate predictions in wild type strains. Typically, evolutionary rationalized objectives like maximization of biomass or minimization of metabolic adjustments are used to predict changes in the flux distribution. However, these objectives become more problematic with an increasing number of gene deletions as the engineered strains have no time to adapt and thus are far from an evolutionary optimum [7].

An alternative way of predicting optimal strain design is to use elementary mode analysis (EMA) [8-13]. EMA allows decomposing a complex metabolic network into unique and biologically meaningful pathways, called elementary modes (EM) [14,15]. An EM is a minimal, and indivisible set of reactions that operates under steady state conditions, while obeying all (ir-)reversibility constraints on the reactions. EM are minimal in the sense that knocking out any one of their contributing reaction will exclude the whole mode from carrying any steady state flux. The entire set of EM, however, describes the full metabolic potential of a cell in an unbiased way. By iteratively deleting EM with unwanted properties a metabolic network of minimal functionality (NMF) can be generated [12]. This procedure, however, does not necessarily return the NMF with the minimum number of deletions.

A rigorous formulation – constrained minimal cut sets (cMCS) – for generating NMF has recently been put forward [16]. It relies on the concept of minimal cut sets (MCS). These are (minimal) sets of deletions, which block undesirable network functionality, like the secretion of unwanted by-products. cMCS allow to keep desirable network properties while simultaneously disabling unwanted functionality [16]. Thus cMCS are ideally suited to design NMF. Moreover, with cMCS it is possible not only to derive the minimal necessary number of metabolic interventions but also to exhaustively predict all possible combinations of deletions resulting in identical NMF.

Here we present an alternative formulation to predict the optimal engineering strategy for the design of MNF. We formulate an optimization problem and show that cMCS can be easily calculated by binary linear programming (BLP) for which commercial and non-commercial solvers are readily available. The scope of our approach is similar to the algorithm presented by [16] but it is more flexible and – most importantly – it allows to include regulatory information in the design process of rational engineering strategies. Static gene regulatory rules can be considered as long as they are formulated in boolean logic terms.

## Theory

### Definitions

We consider the standard steady-state problem of a metabolic network with *m* internal metabolites and *n* reactions, i.e. $S\cdot \hat{v}=0$. Here, ***S*** denotes the *m*×*n* stoichiometric matrix of the network, and $\hat{v}$ the *n*-dimensional flux vector through the network.

Let ***ê*** be an EM flux vector [14,15] fulfilling the steady state condition, and e=e(ê) its binary representation,

(1) $$e_{i}:=e({ê}_{i})=\left\{\begin{array}{c} 1\;\;\text{if}\;{ê}_{i}\neq 0\\ 0\;\;\text{if}\;{ê}_{i}=0 \end{array}\right)\;,\;i=\{1,\ldots ,n\}.$$

*e*_*i*_ indicates whether reaction *i* is part of the EM ***ê***. That is, *e*_*i*_=1 if and only if a reaction is carrying flux either in forward or backward direction. Similar to equation (1) let ***v*** denote the binary representation of any valid flux distribution $\left(\hat{v}\right)$. Then the product

(2) $$e^{T}v\leq e^{T}e=\overset{n}{\underset{i=1}{\sum }}e_{i}^{2}=\overset{n}{\underset{i=1}{\sum }}e_{i}=:||e||,$$

indicates if ***e*** is part of ***v*** as the equality only holds when all “active” reactions in ***e***are also carrying flux in ***v***.

Finally, we group all *q* binary EM of ***S***into three matrices

(3a) $$\begin{array}{ll} G:={\left(e_{1},\ldots ,e_{r}\right)}^{T}, & \; \end{array}$$

(3b) $$\begin{array}{ll} H:={\left(e_{r+1},\ldots ,e_{r+s}\right)}^{T}, & \; \end{array}$$

(3c) $$\begin{array}{ll} K:={\left(e_{r+s+1},\ldots ,e_{r+s+t}\right)}^{T}. & \; \end{array}$$

where *q*=*r* + *s* + *t*, as all EM are in one of the three matrices. The “goal matrix”, ***G***, contains all desirable EM, which define the minimal properties of the NMF and must therefore be kept. The “kill matrix”, ***K***, consists of the unwanted EM, which must not be part of the final flux space and have to be deleted from the network. Finally, the helper matrix, ***H***holds all remaining EM. These modes do not affect the primary design criterion, and therefore may or may not be present in the final design.

In the notation of Hädicke and Klamt [16], our kill matrix ***K*** is their set of target modes **T**. Our ***G***is a subset of their set of desired modes **D**. We collect all other modes in ***H***, while they split these EM between the sets of desired modes, **D**, and the sets of neutral modes. In their formulation Hädicke and Klamt [16] aim to keep at least *n* desired EM out of all modes in **D**. These “surviving” EM build our ***G***. If, however, |**D**|=*n*then **D**=***G***and hence, both definitions are identical.

### Minimum number of deletions, Δ_min_

By setting up a BLP problem, equation (2) may be used to predict the minimal set of knockouts to stop any given set of EM, i.e. the ***K***-matrix, contributing to the steady state flux distribution

(4a) $$\begin{array}{ll} \text{max}\;\;\;\;||x|| & \; \end{array}$$

(4b) $$\begin{array}{ll} \text{s.t.}\;Gx=|g| & \; \end{array}$$

(4c) $$\begin{array}{ll} \;Hx\leq || & \; \end{array}$$

(4d) $$\begin{array}{ll} \;Kx\leq |k|-1 & \; \end{array}$$

(4e) $$\begin{array}{ll} \;x={\left(x_{1},\ldots ,x_{n}\right)}^{T},\;x_{i}\in \{0,1\}\forall i, & \; \end{array}$$

We used ***|g|***=(||*e*_1_||,…,||*e*_*r*_||)^T^, ***|h|***=(||*e*_*r* + 1_||,…,||*e*_*r* + *s*_||)^T^, and ***|k|***=(||*e*_*r* + *s* + 1_||,…,||*e*_*r* + *s* + *t*_||)^T^ to denote the vector of norms of each row of the matrix ***G***,***H***, and ***K***, respectively. ***1***=(1,…,1) represents a vector of ones. The solution vector ***x***, is the binary representation of all reactions participating in the designed NMF. Equation (4) is indeed a BLP problem as ***x*** is binary and $\left(||x||=\overset{n}{\underset{i=1}{\sum }}x_{i}\right)$ is linear.

In equation (4) we used a matrix formulation, which is shorthand for the optimization problem in terms of all *q*=*r* + *s* + *t* binary EM vectors ***e***_*i*_,

$$\begin{array}{lll} \text{max}\; & ||x||\; & \;\\ \text{s.t.}\; & e_{g}^{T}x=||e_{g}||,\;g\in \{1,\ldots ,r\}\; & \;\\ & e_{h}^{T}x\leq ||e_{h}||,\;h\in \{r+1,\ldots ,r+s\}\; & \;\\ & e_{k}^{T}x\leq ||e_{k}||-1,\;\;k\in \;\{r+s+1,\ldots ,r+s+t\}.\; & \; \end{array}$$

Here we used indices *g*,*h*,*k*as a reminder that these EM vectors are the rows of the matrices ***G***,***H***, and ***K***, respectively. Note that each EM acts as a constraint for the optimization problem.

To understand equation (4b) requires that any solution includes all desired EM as – according to equation (2) – only then the product $\left(e_{i}^{T}x\right)$ is limited by the norm of ***e***_*i*_. Similar, equation (4d) demands that its solutions are at least one active reaction short, i.e. has more zeros than any EM in ***K***. As already one single knockout in an EM kills it, these modes will not contribute to the desired design. Finally, constraint (4c) states that the EM of ***H***may be included in the solution. In fact, the inequality (4c) does not constrain the system in any way. Equation (4c) is merely included for the sake of accounting completely for all EM in the network.

The minimal number of deletions can then be determined easily by counting the number of zeros in the calculated solution ***x***,

(5) $$\Delta_{\text{min}}=n-||x||.$$

### Predicting all optimal sets of deletions

Equation (4) may either have no or a finite number of solutions. In the first case, no knockout strategy accommodates all constraints. However, if the constraints are relaxed, i.e. EM are shifted from ***G*** to either ***H*** or ***K*** [the limit being ***G***=(***e***_1_)^T^,***H***=***0***, and ***K***=(***e***_2_,…,***e***_*q*_)^T^], it is always possible to find at least one solution.

Alternate optimal solutions may be found by successively excluding already existing solutions ***x***^(*j*)^ of equation (4) by adding [17,18],

(6a) $$\begin{array}{ll} \underset{i\in B}{\sum }x_{i}-\underset{i\in N}{\sum }x_{i}\leq |B|-1, & \; \end{array}$$

(6b) $$\begin{array}{ll} B=\{i|x_{i}^{\left(j\right)}=1\},\;N=\{i|x_{i}^{\left(j\right)}=0\}. & \; \end{array}$$

Note that repeatedly applying equation (6) will not only generate all sets of different minimal knockouts but also enumerate all other solutions sorted by the number of deletions.

The final sequence contains all possible solutions. It also contains “inefficient” or non-minimal solutions. Consider a series of two reactions, *A*→*B*,*B*→*C*. To suppress the production of *C*, the knocking out of either reaction suffices. Knocking out both is admissible, although inefficient. To avoid calculating non-minimal solutions we split equation (6) into two constraints,

(7a) $$\begin{array}{ll} \underset{i\in B}{\sum }x_{i}\leq |B|-1, & \; \end{array}$$

(7b) $$\begin{array}{ll} \underset{i\in N}{\sum }x_{i}\geq 1. & \; \end{array}$$

In matrix notation these constraints read

(8a) $$\;\begin{array}{ll} {\left[x^{\left(j\right)}\right]}^{T}x & \leq ||x^{\left(j\right)}||-1\; \end{array}$$

(8b) $$\begin{array}{ll} {\left[1-x^{\left(j\right)}\right]}^{T}x & \geq 1.\; \end{array}$$

The first excludes already existing solutions, ***x***^(*j*)^, the second ensures that all solutions will be minimal. In other words, no supersets of already determined solutions will be calculated.

It is possible to influence the succession of solutions by adding weights *w*_*i*_ to the objective function. Rather than maximizing ||***x***|| in equation (4a) we may use

(9) $$\text{max}\;||w^{T}x||,$$

with ***w***^T^=(*w*_1_,…,*w*_*n*_). This allows to easily distinguish chemical from genetic interventions. If uptake reactions are assigned a small and all other reactions a large weight, our algorithm will favor deletions in the uptake reactions as they contribute little to the objective function. Deleting uptake reactions can simply be achieved by removing the substrate from the culture medium. We give guidelines for the choice of reaction weights in the example below.

## Illustrative example

**Figure 1:**
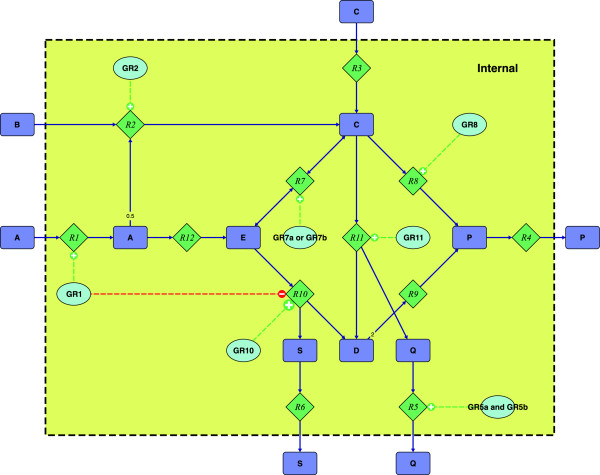
**Illustrative example network.** Illustrative example network containing the metabolites A to E, P, Q and S, the reactions R1 to R12, and the genes GR1, GR2, GR5a, GR5b, GR7a, GR7b, GR8, GR10, and GR11. All reactions are irreversible, except for R7. Transition from E to C is defined as the forward direction of R7. Small numbers in the edges of reactions indicate stoichiometric coefficients, if they are different from one. All metabolites inside the shaded area are considered internal and are subject to the steady state condition. Gene-enzyme-reaction mapping is indicated by dashed lines. Reaction R5 is catalyzed by an enzyme complex encoded by gene GR5a and GR5b. Reaction R7 is catalyzed by two enzymes encoded by GR7a or GR7b. The reaction R10 is catalyzed by GR10. However, activity of R10 is inhibited if GR1 is expressed. For the reaction R3, R4, R6, R9 and R12 no gene-enzyme-reaction mapping is available.

**Figure 2:**
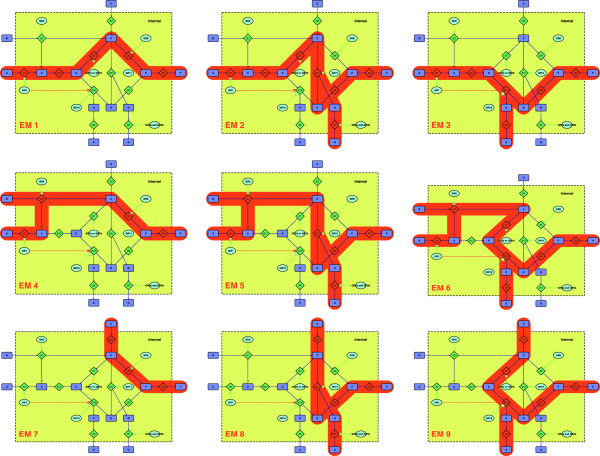
**Illustration of all EM for the example network in Figure **1. The EM are also listed in Table 1.

To illustrate our algorithm we will use the toy network shown in Figure 1. The complete set of EM and their binary representation are listed in Table 1, and illustrated in Figure 2.

**Table 1:** **list of all EM for Figure **1

	**EM flux vector, **$\left({ê}_{i}\right)$													**Binary representation, *e***_***i***_**, of EM flux vector, **$\left({ê}_{i}\right)$													
	**R1**	**R2**	**R3**	**R4**	**R5**	**R6**	**R7**	**R8**	**R9**	**R10**	**R11**	**R12**		**R1**	**R2**	**R3**	**R4**	**R5**	**R6**	**R7**	**R8**	**R9**	**R10**	**R11**	**R12**	$\left(\left||e_{i}|\right|\right)$	
EM 1	1.0	0.0	0.0	1.0	0.0	0.0	1.0	1.0	0.0	0.0	0.0	1.0	***G***=	1	0	0	1	0	0	1	1	0	0	0	1	5	=***|g|***
EM 2	1.0	0.0	0.0	0.5	1.0	0.0	1.0	0.0	0.5	0.0	1.0	1.0	***K***=	1	0	0	1	1	0	1	1	1	0	1	1	8	=***|k|***
EM 3	1.0	0.0	0.0	0.5	0.0	1.0	0.0	0.0	0.5	1.0	0.0	1.0		1	0	0	1	0	1	0	0	1	1	0	1	6	
EM 4	0.5	1.0	0.0	1.0	0.0	0.0	0.0	1.0	0.0	0.0	0.0	0.0		1	1	0	1	0	0	0	1	0	0	0	0	4	
EM 5	0.5	1.0	0.0	0.5	1.0	0.0	0.0	0.0	0.5	0.0	1.0	0.0		1	1	0	1	1	0	0	0	1	0	1	0	6	
EM 6	0.5	1.0	0.0	0.5	0.0	1.0	-1.0	0.0	0.5	1.0	0.0	0.0		1	1	0	1	0	1	1	0	1	1	0	0	7	
EM 7	0.0	0.0	1.0	1.0	0.0	0.0	0.0	1.0	0.0	0.0	0.0	0.0	***H***=	0	0	1	1	0	0	0	1	0	0	0	0	3	=***|h|***
EM 8	0.0	0.0	1.0	0.5	1.0	0.0	0.0	0.0	0.5	0.0	1.0	0.0		0	0	1	1	1	0	0	0	1	0	1	0	5	
EM 9	0.0	0.0	1.0	0.5	0.0	1.0	-1.0	0.0	0.5	1.0	0.0	0.0		0	0	1	1	0	1	1	0	1	1	0	0	6	

List of all EM flux vectors, $\left({ê}_{i}\right)$, and their binary representation, ***e***_*i*_, for the toy network illustrated in Figure 1. EM are sorted by decreasing order of substrate utilization of A. The matrices and vectors ***G***,***H***,***K***, and ***|g|***,***|h|***,***|k|***, respectively, are defined as used in the illustrative example of section “Illustrative example”.

Suppose we use A as feed stock and want to engineer the conversion of A into P. Our aim is to maximize the utilization of A for the efficient production of P. According to Table 1, $\left({ê}_{1}^{T}\right)$ is the only mode which maximizes utilization of A, while efficiently producing P. Hence the goal matrix ***G*** is simply given by ***e***_1_. $\left({ê}_{2}^{T}\right)$, and $\left({ê}_{3}^{T}\right)$ inefficiently synthesize P. $\left({ê}_{4}^{T}\right)$, $\left({ê}_{5}^{\text{T}}\right)$, and $\left({ê}_{6}^{T}\right)$ sub-optimally utilize A. These modes need to be deleted and therefore populate ***K***. The remaining EM do not utilize A. It is irrelevant whether or not those modes are present in the final design as they will have no negative impact. Thus the full BLP problem is defined by the matrices and vectors listed in Table 1. Explicitly, equation (4) reads

$$\max\overset{12}{\underset{i=1}{\sum }}x_{i}$$

subject to

$$\begin{array}{rr} x_{1}+x_{4}+x_{7}+x_{8}+x_{12} & =5\\ x_{3}+x_{4}+x_{8} & \leq 3\\ x_{3}+x_{4}+x_{5}+x_{9}+x_{11} & \leq 5\\ x_{3}+x_{4}+x_{6}+x_{7}+x_{9}+x_{10} & \leq 6\\ x_{1}+x_{4}+x_{5}+x_{7}+x_{8}+x_{9}+x_{11}+x_{12} & \leq 7\\ x_{1}+x_{4}+x_{6}+x_{9}+x_{10}+x_{12} & \leq 5\\ x_{1}+x_{2}+x_{4}+x_{8} & \leq 3\\ x_{1}+x_{2}+x_{4}+x_{5}+x_{9}+x_{11} & \leq 5\\ x_{1}+x_{2}+x_{4}+x_{6}+x_{7}+x_{9}+x_{10} & \leq 6 \end{array}$$

The BLP returns the solution (given in vector notation),

$$x^{\left(1\right)}={\left(\;1\;\;0\;\;1\;\;1\;\;1\;\;1\;\;1\;\;1\;\;0\;\;1\;\;\;1\;\;1\;\right)}^{T}.$$

R2-R9 is the smallest possible MCS to achieve the design criterion. With the solution ***x***^(1)^at hand we use equation (6b) to get the set of indices for the undeleted and deleted reactions, *B*={1,3,4,5,6,7,8,10,11,12}, and, *N*={2,9}, respectively. By adding the constraint equation (7),

$$\begin{array}{lll} x_{1}+x_{3}+x_{4}+x_{5}+x_{6}+x_{7}+x_{8}+x_{10}+x_{11}+x_{12} & \leq 9,\; & \;\\ x_{2}+x_{9} & \geq 1,\; & \; \end{array}$$

to the equations above and resolving the problem, an alternative MCS may be calculated. An overview of all MCS is given in Table 2.

**Table 2:** **List of all MCS for Figure **1

							***w***_**1**_								***w***_**2**_
						***i***	**minimal cut set**			***f*_*i*_**		**minimal cut set**	***f*_*i*_**		
						1	R2	R9		9.0		R2	R5	R10	301.2	*
						2	R2	R5	R6	8.0		R2	R10	R11	301.2	*
						3	R2	R5	R10	8.0	*	R2	R9		204.2	
						4	R2	R6	R11	8.0		R2	R5	R6	203.2	
						5	R2	R10	R11	8.0	*	R2	R6	R11	203.2	

List of all MCS for the most efficient production of P from A in the network Figure 1. Two different weight vectors were used, $\left(w_{1}^{T}\right)$ = (1 1 1 1 1 1 1 1 1 1 1 1), and $\left(w_{2}^{T}\right)$ = (0.1 0.1 0.1 99 1 99 2 1 99 1 1 99). MCS are sorted in decreasing order of the objective function $\left(f_{i}=w_{j}^{T}x^{\left(i\right)}\right)$, *j* = {1,2} as calculated by our algorithm. (The sequence of MCS with equal objective value may differ depending on the BLP algorithm.) * marks MCS for which full genetic information is available.

The calculated solutions do not take gene-enzyme-reaction mapping into account. As indicated in Figure 1, implementing the smallest MCS (R2 and R9) is infeasible, due to missing genetic information for R9. To account for biological feasibility we reevaluate the BLP problem using the weight function,

$$w_{2}^{T}=(\;0.1\;\;0.1\;\;0.1\;\;99\;\;1\;\;99\;\;2\;\;1\;\;99\;\;1\;\;1\;\;99\;).$$

Here we assigned small weights (0.1) to uptake reactions (R1 to R3), which are easy to “delete” by removing the corresponding substrate from the growth medium. Reactions with missing genetic information (R4, R6, R9, R12) received high weights (99), which made them “harder” to delete. Note that R3 is also lacking genetic information. Since it is an easily “deletable” uptake reaction, R3 was weighted with 0.1 rather than 99. We associated R7 with a weight of two as this reaction is catalyzed by two independent enzymes. On the other hand, R5 retained its weight of one as the reaction is catalyzed by a single enzyme complex encoded by two genes. The sequence of all possible MCS is listed in Table 2. Note that by using weight functions, experimentally implementable engineering strategies are predicted first. All other solutions are predicted, too. However, the weight function is able to account for experimental difficulties in implementing a reaction deletion *in vivo.*

In general, we assign reaction weights according to the number of independent enzymes or enzyme complexes catalyzing a reaction in parallel. Uptake reactions, however, should be favored over genetic deletions. Therefore the sum of all weights for uptake reactions should be smaller than the smallest weight of the non-uptake reactions. On the other hand the weight for a “non-deletable” reaction (i.e. a reaction without genetic information) should be larger than the sum of all other “deletable” reactions.

## Including regulation

In the following we demonstrate the inclusion of boolean regulation by way of example. Typically, regulatory information is represented in logic statements [19] which may readily be added to equation (4). In Figure 1 we illustrate typical gene-enzyme-reaction mappings, like reactions catalyzed by single enzymes (G↦R), by multiple enzymes in parallel [(Ga OR Gb)↦R], or by single enzyme complexes [(Ga AND Gb)↦R]. As demonstrated, these interactions may be incorporated in weight functions. By adding appropriate constraints, BLP also allows the integration of inhibitions, like (NOT G)↦R; [Ga AND (NOT Gb)]↦R. For example a single gene-enzyme-reaction mapping, G↦R, is easily converted into the BLP constraint, *G*−*R*=0. Similarly, the negation (NOT G)↦R transforms into *G* + *R*=1. In Table 3 we summarize other interactions along with their constraint based formulation. An extension to more interaction partners is straight forward. More specifically, we list the regulatory constraints for the network in Figure 1 in Table 4.

**Table 3:** Truth table for the conversion of regulatory functions into constraints for BLP

			**Function / constraint**			
***G*_*a*_**	***G*_*b*_**	***R***	**(Ga OR Gb)↦R /**	**[ (NOT Ga) OR Gb ]↦R /**	**(Ga AND Gb)↦R /**	**[ (NOT Ga) AND Gb]↦R /**
			**−1≤*G*_*a*_ + *G*_*b*_−2*R*≤0**	**0≤*G*_*a*_ + *G*_*b*_−2*R*≤1**	**0≤*G*_*a*_ + *G*_*b*_−2*R*≤1**	**−1≤−*G*_*a*_ + *G*_*b*_−2*R*≤0**
0	0	0	0	*	0	0
0	0	1	*	-2	*	*
0	1	0	*	*	1	*
0	1	1	-1	-1	*	-1
1	0	0	*	-1	1	-1
1	0	1	0	*	*	*
1	1	0	*	*	*	0
1	1	1	0	-2	0	*

^*^Marks values outside the constraint range.

**Table 4:** **Regulatory constraints in Figure **1 for use in BLP

	
*y*_1_−*x*_1_= 0	*y*_5*a*_ + *y*_5*b*_−2*x*_5_≤ 1
−*y*_1_ + *y*_10_−2*x*_10_≥−1	*y*_7*a*_ + *y*_7*b*_−2*x*_7_≥−1
−*y*_1_ + *y*_10_−2*x*_10_≤ 0	*y*_7*a*_ + *y*_7*b*_−2*x*_7_≤ 0
*y*_2_−*x*_2_= 0	*y*_8_−*x*_8_= 0
*y*_5*a*_ + *y*_5*b*_−2*x*_5_≥ 0	*y*_11_−*x*_11_= 0

Regulatory constraints for use in BLP for the metabolic network in Figure 1. Here *x*_*i*_, and *y*_*j*_ denote reactions and genes, respectively.

Adding the regulatory constraints in Table 4 we maximize the BLP problem equation (4) using $\left(\left||{\text{w}}_{2}^{\text{T}}x||+||y|\right|\right)$ as objective. Here

$$y={\left(\;y_{1}\;\;y_{2}\;\;y_{5a}\;\;y_{5b}\;\;y_{7a}\;\;y_{7b}\;\;y_{8}\;\;y_{10}\;\;y_{11}\;\right)}^{\text{T}}$$

denotes the binary vector of the involved genes. Note that integrating regulation into our algorithm only requires additional constraints and an extended objective function. This is in contrast to the original cMCS-method [16]. cMCS requires an independent, separate preprocessing step first to identify and remove all EM, which are in contradiction to regulatory constraints. Only then, cMCS can be applied. BLP, however, allows simultaneously integrating stoichiometric and regulatory constraints in a unified framework. Moreover, BLP allows to fully consider reconstructed transcriptional regulatory networks.

Note that by using $\left(\left||w_{2}^{\text{T}}x||+||y|\right|\right)$ as objective, we optimize for the combined effect of both, reactions and genes. Thus our objective predicts interventions with the smallest overall impact first. Again, it is possible to influence the succession of solutions by using weight functions for genes as well. However, this has not been investigated.

In Table 5 we collect all MCS to the regulatory BLP problem for the network in Figure 1. Note that the MCS 1 and 2 do not differ in terms of reactions but in terms of the deleted genes. All feasible MCS require two deletions at the genetic level, but three reaction deletions. The third reaction (R10) is suppressed due to GR1, rather than deleted. According to the design criterion GR1 is expressed in all desired EM. Thus all solutions to the BLP problem will necessarily be characterized by a down regulated R10. This reduces the total number of different MCS (in terms of reactions) from five to three (compare Table 2 and Table 5). Note that the MCS R2-R5-R6 and R2-R6-R11 of Table 2 are not MCS for the regulated system. As in the regulated system R10 is always suppressed, deletion of R6 becomes redundant. For the regulated network R2-R5-R6 and R2-R6-R11 are only cut sets, rather than MCS.

**Table 5:** **List of all MCS for the regulatory BLP in Figure **1

***i***	**Gene deletion**		**Reaction deletion**			***f*_*i*_**	
1	GR2	GR5a	R2	R5	R10	308.2	*
2	GR2	GR5b	R2	R5	R10	308.2	*
3	GR2	GR11	R2	R10	R11	308.2	*
4	GR2		R2	R9	R10	211.2	

List of all MCS for the regulatory BLP. MCS are sorted in decreasing order of the objective function $\left(f_{i}=w_{2}^{T}x^{\left(i\right)}+||y^{\left(i\right)}||\right)$ as calculated by our algorithm. (The sequence of MCS with equal objective value may differ depending on the BLP algorithm.) * marks MCS for which full genetic information is available. MCS are split in the gene deletion part and the reaction deletion part. Note that the first three MCS require deletions of two genes. The corresponding reaction deletions are a consequence of those deletions. MCS 4 however, is not fully annotated (noticeable in the drop of *f *_*i*_), and would require the deletions of genes and reactions (GR2 and R9).

## Optimizing metabolic functionality

All solutions to equation (4) and (6) are characterized by the smallest possible number of knockouts. However, their metabolic functionality may differ. This can be the case if ***H***≠***0***, as individual EM from the helper matrix may be added or removed. With all optimal solutions at hand it is easy to pick those which additionally optimize the number of “surviving” EM. That is, we may look for solutions with the smallest/largest set of EM contributing to the metabolic functionality. However, for these questions it is not necessary to fully enumerate all solutions of equation (4). The answer is accessible by BLP as well.

Let

(10) $$p(e)=\underset{i\in C}{\prod }e_{i},\;C=\{i|e_{i}=1\},$$

be the product of all reactions contributing to an EM. *p*(***e***)=0 if any reaction contributing to the EM ***e***_*i*_ is knocked out, and 1 otherwise. Thus *p*(***e***) indicates whether an EM contributes to the final steady state. Optimizing the number of surviving EM means we maximize (minimize) the number of participating EM,

(11) $$\text{min/max}\underset{i}{\sum }p(e_{i}),\;i=\{r+1,\ldots ,r+s\}.$$

Here *i* runs over all EM which may contribute to the steady state, i.e. over all modes stored in ***H***. Although *p*_*i*_=*p*(***e***_*i*_) is a product of binary variables, it is convertible into BLP using standard transformation rules [20] yielding

(12a) $$\begin{array}{ll} \text{min/max}\;\;\;\;||p|| & \; \end{array}$$

(12b) $$\begin{array}{ll} \text{s.t.}\;Hx\geq p.*|h| & \; \end{array}$$

(12c) $$\begin{array}{ll} \;Hx\leq p+|h|-1 & \; \end{array}$$

(12d) $$\begin{array}{ll} \;p={\left(p_{r+1},\ldots ,p_{r+s}\right)}^{T},\;\in \{0,1\}\forall i & \; \end{array}$$

(12e) $$\begin{array}{ll} \;\text{equation (4b) to equation (4e)}, & \; \end{array}$$

where we used the MATLAB notation for array multiplication “.∗” to denote the element-wise product of the vectors ***p***and ***|h|***.

Suppose that the kill matrix ***K***and ***H*** contain all EM of a metabolic system, i.e. ***G***=***0***. Then equation (12) allows to determine the maximum number of surviving EM. It is interesting to connect this result to the original formulation of the cMCS approach [16]. In their paper the authors define an intervention problem “by a set **T** of target modes and a set **D** of desired modes of which at least *n* must not be hit by a cMCS” [16]. Here, their **T** corresponds to our ***K***, while the row vectors of ***H***will in general be a superset of **D**. However, for any **T** equation (12) gives an upper bound to the preserved number *n* of desired EM, which is an important parameter in the cMCS-formulation.

## Result

### Realistic example

In analogy to [16] we validated our approach by predicting MCS for the efficient production of ethanol in *E. coli* using data presented by [12]. There, the authors used a small-scale metabolic model under anaerobic conditions, calculated all its 5,010 EM, optimized for the most efficient production of ethanol from glucose, and came up with a strain design where seven reactions were removed from the network. They found that only twelve EM contributed to the optimal design. All of them produced ethanol and four EM were also growth coupled. (The full model used by [12] is listed in the Additional file 1: Table S1.)

Using our algorithm we were able to design a cell with identical functional capabilities, but with fewer knockouts. In fact, the minimally necessary number of reaction deletions was six [consistent with identical findings in [16]]. In our simulation ***G*** consisted of the twelve optimal EM identified by [12], ***H***=***0***, and ***K***contained the remaining 4,998 EM. In less than 25 sec computation time we found 1,048 MCS of which 252 required exactly seven deletions. One of these MCS was the solution given by [12]. Again, our findings are identical with [16]. However, note that 71% of these 1,048 MCS, are not deletable due to missing annotations or are in principle undeletable. We used the gene-enzyme-reaction mapping as given by [12], who annotated only enzymatic reactions, but no transporters. Here, we consider all non-annotated reactions in the model of [12] as “undeletable”. Most of these non-annotated reactions are transport reactions. Some of them may merely miss an annotation, and – in principle – could be deleted. Others however, are diffusion transporters and cannot be blocked. For simplicity, we do not distinguish between these two types and considered both as undelatable. In contrast however, we do consider uptake reactions as deletable – independent of any possible annotation – as these transporters are simply “deletable” by removing the substrate form the medium.

By using a weight function (one possible function is given in the Additional file 1: Table S2) our algorithm is able to predict biologically feasible deletions first. In fact, the *in vivo *implementation of the smallest, fully annotated, biologically feasible MCS requires seven gene deletions. We found eight alternate MCS. In comparison, the experimentally implemented strain by [12] had eight knockouts.

To test the robustness of the alternate optimal solutions against variation in the weight vector, we randomly changed each weight in the range between ± 20% and repeated our calculation 1,000 times. Every time we found the same eight solutions with seven deletions. To further test the stability of our predictions, we incrementally changed each weight in such a way that after 150 steps all weights are one and thus recover the situation without weights (see the Additional file 1: Figure S1). Even with this procedure we find stable predictions over a wide range of different weights. (For details on the procedure and specific results we refer to the Additional file 1: section “Robustness of optimal solutions against variations in the weight vector” and Additional file 1: Figure S1.)

However, even with a weight function it is possible to fully enumerate all solutions.

To test wether our algorithm is able to handle larger system we repeated the analysis with the full model used by [12], that is, without restricting the model to glucose uptake under anaerobic conditions first. The complete model contained 429,276 EM – including the elementary futile cycle succinate dehydrogenase and its reverse reaction fumarate reductase (reactions R_TCA10 and R_TCA7 in the model). This cycle was disregarded in the following analysis.

Again, we used the same twelve EM (identified by Trinh *et. al.*[12] and defined above) as design criterion. (That is, the goal matrix ***G*** consisted of the twelve optimal EM, ***H***=***0***, and ***K***contained the remaining EM.) Without any weight function and additional constraints, at least eleven reaction deletions are required to reach the design goal. In total we found 55,488 MCS, 1.440 of which require the minimal number of eleven reaction deletions. (For the sake of completeness we listed the maximal number of MCS as function of deletions in the Additional file 1: Table S3.) Note, however, that these deletions are knockouts of reactions without regard to biological feasibility. In fact we found that none of those 1.440 MCS are fully annotated. Furthermore, only 27.7% of all 55,488 MCS are fully annotated, enzymatic reactions. In all other MCS at least one reaction was a transport reaction, for which genetic information was lacking. In order to calculate biologically feasible solutions first we included the weight function given in the Additional file 1: Table S2.

Using the weight function listed in the Additional file 1: Table S2, at least twelve reaction deletions were required to reach the design goal. Out of these twelve deletions five are uptake reactions (L-arabinose, D-galactose, D-mannose, D-xylose, and oxygen). Removing these substrates from the growth medium recovers the initial model discussed above: anaerobic growth with glucose as the sole carbon source. We found eight equivalent solutions. Those solutions are exactly the solutions predicted for the anaerobic model above.

In their paper [12] the authors noted that six out of the twelve EM in the optimal design are inactive. Those six, inactive EM use the pyruvate dehydroganese complex (reaction R_GG13) which is down regulated under anaerobic conditions (reaction R_TRA11 = 0) [12,21]. Additionally the repression of the glyoxylate shunt (reaction R_GLB1) during growth on glucose (reaction R_GG1) [22,23] has not been considered in the above analysis. With our algorithm, however, it is possible to simply include these regulatory information in the form of two additional constraints, R_GG1 + R_GLB1 ≤ 1, and R_TRA11 + R_GG13 ≤ 1. Note however, an analysis which combines the regulatory information with the previous design goals (i.e. those twelve desired EM) does not yield a solution as the desired design and the regulatory constraints are inconsistent.

We repeated the analysis, included weights and regulatory constraints, and used the six potentially active EM as goal matrix. We predicted 13 deletions. Two of which (R_GLB1 and R_GG13) are, however, no deletions, but in fact the result of the regulation. As expected, we found that all MCS were characterized by a down-regulation of R_GLB1 and R_GG13.

## Discussion

Elementary mode analysis has been identified as a promising tool for metabolic engineering. However, the analysis of millions or billions of EM still poses difficulties. Recently [16] introduced the concept of cMCS, which allows calculating all optimal metabolic engineering strategies. Here we showed that equivalent results can be obtained by simple integer programming.

We partitioned EM into three categories: goal modes, kill modes, and helper modes. The first group contains the desired functionality. All modes in this group will also be present in the final NMF. Kill modes, on the other hand, are all those modes which will definitely get deleted from the network. The third and final group collects all other modes, which may or may not be present in the final design. With respect to the design criterion this last group of modes neither contributes to nor counteracts the design goal. We then reformulated the problem of calculating MCS to generate NMF as a linear optimization problem. Our approach is very intuitive and structurally reminiscent to ordinary flux balance analysis. The matrix of EM replaces the stoichiometric matrix. Constraints are not set by the mass-balance but by design requirements. More specifically, binarized EM show up as constraints on the admissible flux space. By optimizing the admissible flux space, maximal or minimal intervention strategies (with respect to the number of deletions) can be predicted.

In the case of optimal ethanol production we demonstrated that BLP is able to give identical results as compared to cMCS [16]. In fact, in a special case our method is formally equivalent to cMCS (see section “Definitions”). Moreover, by optimizing metabolic functionality BLP allows to calculate an upper bound for the maximum number of persevered EM, which is an important parameter in the cMCS-formulation. The two methods differ in that BLP uses a fixed set of desirable EM – the goal matrix ***G*** –, while in cMCS EM are chosen automatically form a pool of desirable EM, **D**, such that at least *n* modes survive. However, if the surviving modes are known an identical BLP problem can be set up.

The major advantage of our reformulation is its easy integration of (binary) transcriptional regulation. Regulatory information may simply be included as additional constraints. We have shown that our formulation allows a regulatory coupling between reactions and between genes and reactions alike. In Table 4 and in Table 3, we listed several examples for simple regulatory interactions. However, these expressions are easily expandable to more complex functions. The mapping between genes, proteins and reactions, as well as transcriptional regulation can be included as long as they are formulated as static boolean constraint. At least for well studied organisms like *E. coli* and *S. cerevisiae* curated transcriptional regulatory networks are readily available [24]. However, dynamic regulations or cyclic causalities pose immense difficulties and cannot be represented in our approach.

Additionally the BLP formulation offers more flexibility in the way solutions are predicted. By using weights in the objective function it is possible to account for experimental difficulties in the implementation of the strain. This allows to prioritize biologically feasible MCS over infeasible ones and – in contrast to other, optimized based methods [9] – does not effect the ability to calculate the complete set. Taking biological feasibility into account seems advantageous as in our example we have demonstrated that due to the lacking gene-enzyme-reaction mapping roughly 70% of the predicted solutions would require the deletion of at least one non-enzymatic reaction. Due to the combinatorial explosion of the number of EM [25], we expect that the percentage of unrealizable solutions is increasing further with augmenting system size. Obviously, sorting of solutions with respect to biological feasibility can be done in a separate post-processing step, too. However, in our implementation we get the sorting for “free”, i.e. without any additional computational steps. At least in the case of *E. coli* we demonstrated that our predictions are robust against variations in the weights. In particular we found that our choice of weights is very conservative and far from the limits detected in the robustness analysis (see the Additional file 1: Figure S1). It may be possible to integrate a weighting function in the algorithm presented by [16] as well. However, it has not been demonstrated yet.

**Figure 3:**
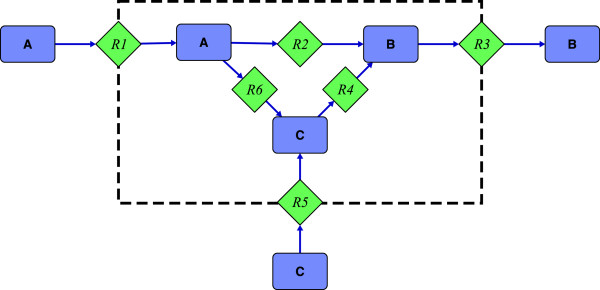
**Illustrative toy network.** Illustrative toy network containing the metabolites A to C, and the reactions R1 to R6. All reactions are irreversible. The area inside the dashed box indicates the “cell interior”. The network consists of three EM: R1-R2-R3, R1-R6-R4-R3, and R5-R4-R3.

Part of the flexibility of our approach is its ability to optimize metabolic functionality. This can be easily demonstrated in a simple example as illustrated in Figure 3. The network consists of three EM. Lets suppose that R1-R2-R3 is the only desirable EM (***G***= R1-R2-R3, ***H***= R5-R4-R3, and ***K***= R1-R6-R4-R3). A NMF can be easily generated by either knocking out either R4 or R6. The metabolic functionality of the resulting networks, however, differs significantly. By deleting R4 the only “surviving” mode is the desired goal mode. Thus the network is in fact a NMF, as no other functionality is available. On the other hand, by deleting R6 the network still has the desired properties, but retains additional functionality (conversion of C to B) without compromising the original design criterion. Alternatively we may define a kill-matrix ***K*** and calculate the resulting network of minimal or maximal functionality. BLP is able to predict solutions with the smallest/largest set of EM contributing to the metabolic functionality and distinguish between those two extremes without enumerating the full solution space. This feature therefore opens a way to include secondary objectives in the design process.

An integer programming problem sits at the heart of our algorithm. Integer programs are inherently difficult to solve [20]. Nevertheless efficient commercial and non-commercial solvers are available. Still the question remains if BLP is fit for solving even larger problems than the one presented. Even with current technologies a complete EMA can only be done for small-scale problems, typically involving about 100 reactions. These 100 reactions transform into 100 binary variables in the BLP problem. Their handling is easy [20]. On the other hand, the number of EM in metabolic networks explodes combinatorially with the system size [25], which translates into millions and even billions of constraints for BLP. These constraints are highly redundant and can be efficiently compressed using various preprocessing techniques typically already included in available solvers (or various preprocessing methods see [20,26]). For instance, the initial BLP problem to predict the smallest MCS in the full *E. coli* model [12] (see above) contained 429,276 constraints for 71 variables. After preprocessing we transformed the problem into 28 constraints for 34 variables, which dramatically improved the computational performance (data not shown). The compression is also beneficial in the context of the original formulation of cMCS. The problem may be set up as integer program first, followed by standard preprocessing. The reduced problem may then be solved by the adapted Berge algorithm presented by [16].

We tested various off-the-shelf software packages to solve the BLP problem. The implementation of our algorithm merely required setting up the input parameters for those solvers. We found that our approach is computationally modest and scalable. In fact, we were able to successfully repeat the analysis for the much larger core metabolic network of [24] with its 271 million EM on a standard personal computer. We used the single most efficient EM for the production of ethanol form glucose as design criterion (all other modes were killed). In 122 sec our algorithm found all 2,304 MCS with the minimum number of 26 deletions. (The total program runtime, which included reading all EM from disk and calculating the MCS, was 10 min 30 sec.) The problem here, and with cMCS in general, is not the handling of millions of EM (although data handling required 80% of the total runtime), but to calculate these modes in the first place [16]. However, promising results on efficiently enumerating the full set of EM have recently been published [27,28].

## Conclusion

In summary, we have demonstrated an efficient and easy to implement method to rationally predict engineering strategies for the improvement of production hosts. Optimal pathways were identified using elementary mode analysis. Based on integer/binary programming we were then able to predict all minimal intervention strategies to design a strain with desirable metabolic capabilities. Our method is based on the concept of constrained minimal cut sets, but offers much more flexibility in the prediction of engineering targets, including most prominently the possibility of easily integrating gene regulation.

## Methods

We used *efmtool*[28] to calculate the complete set of EM for a network and Gurobi Optimizer 5.0, http://www.gurobi.com/ for solving the BLP problem. *efmtool* is open source and freely available; Gurobi offers a free academic license.

## Abbreviations

BLP: Binary linear programming; cMCS: Constrained minimal cut set; EM: Elementary modes; EMA: Elementary mode analysis; MCS: Minimal cut set.

## Competing interests

We declare that we have no competing interests.

## Authors’ contributions

CJ and JZ designed, analyzed and wrote the paper. Both authors read and approved the final manuscript.

## Supplementary Material

Additional file 1 **Supplementary material.** A pdf containing all additional data, figures and tables. Click here for file
