# Supplementary material for: Designing optimal cell factories: integer programming couples elementary mode analysis with regulation
Source: BMC Syst Biol. 2012 Aug 16;6:103. doi: 10.1186/1752-0509-6-103 (PMC3560272; doi:10.1186/1752-0509-6-103)
Supplement: Additional file 1 — Supplementary material. A pdf containing all additional data, figures and tables. [file 1752-0509-6-103-S1.pdf]

SUPPLEMENTARY MATERIAL TO "DESIGNING OPTIMAL CELL FACTORIES: INTEGER PROGRAMING COUPLES ELEMENTARY MODE ANALYSIS WITH REGULATION"

Table S1. Metabolic core model of *E. coli*'s central metabolism (Trinh *et al.*, 2008). Cytoplasmic metabolites were assumed to be in steady state, all others were considered to be external.

ID	Stoichiometric reaction	Genes
R_ANA1	M_co2_c + M_pep_c → M_oaa_c	ppc
R_ANA2	M_mal_L_c + M_nad_c → M_co2_c + M_nadh_c + M_pyr_c	sfcA, maeB
R_ANA3	M_atp_c + M_oaa_c → M_adp_c + M_co2_c + M_pep_c	pckA
R_ARA1	M_ara_L_c + M_atp_c → M_adp_c + M_xu5p_D_c	araA, araB, araD
R_BIO	1.642 M_3pg_c + 1.207 M_accoa_c + 40.680 M_atp_c + 0.512 M_e4p_c + 0.017 M_f6p_c + 0.031 M_g3p_c + 0.049 M_g6p_c + 4.079 M_nad_c + 18.320 M_nadph_c + 2.355 M_oaa_c + 0.960 M_pep_c + 3.920 M_pyr_c + 0.860 M_r5p_c + 1.426 M_akg_c + 12.502 M_nh4_c → 40.680 M_adp_c + 1.207 M_coa_c + 4.079 M_nadh_c + 18.320 M_nadp_c	
R_EDP1	M_6pgc_c → M_dd6pg_c	edd
R_EDP2	M_dd6pg_c → M_g3p_c + M_pyr_c	eda
R_FC1r	M_nad_c + M_nadph_c ⇌ M_nadh_c + M_nadp_c	pntAB
R_FC2	M_amp_c + M_atp_c ⇌ 2 M_adp_c	adk
R_FEM1	M_coa_c + M_pyr_c → M_accoa_c + M_for_c	pflB, tdcE
R_FEM2	M_pyr_c + M_q8_c → M_ac_c + M_co2_c + M_q8h2_c	poxB
R_FEM3	M_nadh_c + M_pyr_c → M_lac_D_c + M_nad_c	ldhA
R_FEM4	M_for_c → M_co2_c + M_h2_b	hycBCDEFG, fdhF
R_FEM5	M_accoa_c + M_nadh_c → M_acald_c + M_coa_c + M_nad_c	adhE
R_FEM6	M_acald_c + M_nadh_c → M_etoh_c + M_nad_c	adhP, adhE, adhBZM
R_FEM7	M_accoa_c → M_coa_c + M_actp_c	pta
R_FEM8	M_actp_c + M_adp_c → M_ac_c + M_atp_c	ackAB
R_FEM9	M_pyr_c → M_acald_c + M_co2_c	pdeZM
R_GAL1	M_atp_c + M_gal_c → M_adp_c + M_g6p_c	galK, pgm
R_GG1	M_glc_D_b + M_pep_c → M_g6p_c + M_pyr_c	ptsG, ptsHI, crr
R_GG2r	M_g6p_c ⇌ M_f6p_c	pgi
R_GG3	M_atp_c + M_f6p_c → M_adp_c + M_fdp_c	pfkA, pfkB
R_GG4	M_fdp_c → M_f6p_c	glpX, fbp
R_GG5r	M_fdp_c ⇌ M_dhap_c + M_g3p_c	fbaB, fbaA
R_GG6r	M_dhap_c ⇌ M_g3p_c	tpiA
R_GG7r	M_g3p_c + M_nad_c ⇌ M_13dpg_c + M_nadh_c	gapA
R_GG8r	M_3pg_c + M_atp_c ⇌ M_13dpg_c + M_adp_c	pgk
R_GG9r	M_2pg_c ⇌ M_3pg_c	pgml, ytjC, pgmA
R_GG10r	M_2pg_c ⇌ M_pep_c	eno
R_GG11	M_adp_c + M_pep_c → M_atp_c + M_pyr_c	pykA, pykF
R_GG12	M_atp_c + M_pyr_c → M_amp_c + M_pep_c	pps
R_GG13	M_coa_c + M_nad_c + M_pyr_c → M_accoa_c + M_co2_c + M_nadh_c	lpdA, aceEF
R_GLB1	M_icit_c → M_glx_c + M_succ_c	aceA
R_GLB2	M_accoa_c + M_glx_c → M_coa_c + M_mal_L_c	glcB, aceB
R_MAN1	M_atp_c + M_man_b → M_adp_c + M_m6p_c	manXYZ, ptsHI, crr
R_MAN2	M_m6p_c → M_f6p_c	manA
R_OPM1	2 M_adp_c + M_nadh_c + M_o2_c → 2 M_atp_c + M_nad_c	nuoAHJKLMNE, FGBCI, atpABCDEFGHI
R_OPM2	M_adp_c + M_o2_c + M_q8h2_c → M_atp_c + M_q8_c	cyoABCD, atpABCDEFGHI
R_OPM3	M_atp_c → M_adp_c + M_atpmain_b	
R_OPM4r	M_nadh_c + M_q8_c ⇌ M_nad_c + M_q8h2_c	ndh
R_PPP1	M_g6p_c + M_nadp_c → M_6pgl_c + M_nadph_c	zwf
R_PPP2	M_6pgl_c → M_6pgc_c	pgl

Continued on next page

Table S1. Continued from previous page

ID	Stoichiometric reaction	Genes
R_PPP3	M_6pgc_c + M_nadp_c → M_co2_c + M_nadph_c + M_ru5p_D_c	gnd
R_PPP4r	M_ru5p_D_c ⇌ M_xu5p_D_c	rpe
R_PPP5r	M_r5p_c ⇌ M_ru5p_D_c	rpiA, als1
R_PPP6r	M_r5p_c + M_xu5p_D_c ⇌ M_g3p_c + M_s7p_c	tktAB
R_PPP7r	M_g3p_c + M_s7p_c ⇌ M_e4p_c + M_f6p_c	talAB
R_PPP8r	M_e4p_c + M_xu5p_D_c ⇌ M_f6p_c + M_g3p_c	tktAB
R_TCA1	M_accoa_c + M_oaa_c → M_cit_c + M_coa_c	prpC, gltA
R_TCA2r	M_cit_c ⇌ M_acon_C_c	acnAB
R_TCA3r	M_acon_C_c ⇌ M_icit_c	acnAB
R_TCA4	M_icit_c + M_nadp_c → M_akg_c + M_co2_c + M_nadph_c	icd
R_TCA5	M_akg_c + M_coa_c + M_nad_c → M_co2_c + M_nadh_c + M_succoa_c	lpdA, sucAB
R_TCA6r	M_atp_c + M_coa_c + M_succ_c ⇌ M_adp_c + M_succoa_c	sucCD
R_TCA7	M_q8_c + M_succ_c → M_fum_c + M_q8h2_c	sdhABCD
R_TCA8r	M_fum_c ⇌ M_mal_L_c	fumABC
R_TCA9r	M_mal_L_c + M_nad_c ⇌ M_nadh_c + M_oaa_c	mdh
R_TCA10	M_fum_c + M_q8h2_c → M_q8_c + M_succ_c	frdABCD
R_TRA1	M_etoh_c → M_etoh_b	
R_TRA2	M_ac_c → M_ac_b	
R_TRA3	M_nh4_b → M_nh4_c	
R_TRA4	M_lac_D_c → M_lac_D_b	
R_TRA5	M_succ_c → M_succ_b	
R_TRA6	M_for_c → M_for_b	
R_TRA7	M_co2_c → M_co2_b	
R_TRA8	M_atp_c + M_xylo_b → M_adp_c + M_xylo_c	xylGHF
R_TRA9	M_atp_c + M_gal_b → M_adp_c + M_gal_c	mglABC
R_TRA10	M_atp_c + M_ara_L_b → M_adp_c + M_ara_L_c	araFGH
R_TRA11	M_o2_b → M_o2_c	
R_XYL1	M_xylo_c → M_xylu_c	xylA
R_XYL2	M_atp_c + M_xylu_c → M_adp_c + M_xu5p_D_c	xylB

ID	Metabolite name	Compartment
M_13dpg_c	3-Phospho-D-glyceroyl-phosphate	Cytoplasm
M_2pg_c	D-Glycerate-2-phosphate	Cytoplasm
M_3pg_c	3-Phospho-D-glycerate	Cytoplasm
M_6pgc_c	6-Phospho-D-gluconate	Cytoplasm
M_6pgl_c	6-phospho-D-glucono-1-5-lactone	Cytoplasm
M_ac_b	Acetate	Extracellular
M_ac_c	Acetate	Cytoplasm
M_acald_c	Acetaldehyde	Cytoplasm
M_accoa_c	Acetyl-CoA	Cytoplasm
M_acon_C_c	cis-Aconitate	Cytoplasm
M_actp_c	Acetyl-phosphate	Cytoplasm
M_adp_c	ADP	Cytoplasm
M_akg_c	2-Oxoglutarate	Cytoplasm
M_amp_c	AMP	Cytoplasm
M_ara_L_b	L-Arabinose	Extracellular
M_ara_L_c	L-Arabinose	Cytoplasm
M_atp_c	ATP	Cytoplasm
M_atpmain_b	ATP	Extracellular
M_cit_c	Citrate	Cytoplasm
M_co2_b	CO2	Extracellular
M_co2_c	CO2	Cytoplasm

Continued on next page

Table S1. Continued from previous page

ID	Metabolite name	Compartment
M_coa_c	Coenzyme-A	Cytoplasm
M_dd6pg_c	2-Dehydro-3-deoxy-6-phospho-D-gluconate	Cytoplasm
M_dhap_c	Dihydroxyacetone-phosphate	Cytoplasm
M_e4p_c	D-Erythrose-4-phosphate	Cytoplasm
M_etoh_b	Ethanol	Extracellular
M_etoh_c	Ethanol	Cytoplasm
M_f6p_c	D-Fructose-6-phosphate	Cytoplasm
M_fdp_c	D-Fructose-1-6-bisphosphate	Cytoplasm
M_for_b	Formate	Extracellular
M_for_c	Formate	Cytoplasm
M_fum_c	Fumarate	Cytoplasm
M_g3p_c	Glyceraldehyde-3-phosphate	Cytoplasm
M_g6p_c	D-Glucose-6-phosphate	Cytoplasm
M_gal_b	D-Galactose	Extracellular
M_gal_c	D-Galactose	Cytoplasm
M_glc_D_b	D-Glucose	Extracellular
M_glx_c	Glyoxylate	Cytoplasm
M_h2_b	h2_b	Extracellular
M_icit_c	Isocitrate	Cytoplasm
M_lac_D_b	D-Lactate	Extracellular
M_lac_D_c	D-Lactate	Cytoplasm
M_m6p_c	D-Mannose 6-phosphate	Cytoplasm
M_mal_L_c	L-Malate	Cytoplasm
M_man_b	D-Mannose	Extracellular
M_nad_c	Nicotinamide-adenine-dinucleotide	Cytoplasm
M_nadh_c	Nicotinamide-adenine-dinucleotide-reduced	Cytoplasm
M_nadp_c	Nicotinamide-adenine-dinucleotide-phosphate	Cytoplasm
M_nadph_c	Nicotinamide-adenine-dinucleotide-phosphate-reduced	Cytoplasm
M_nh4_b	Ammonium	Extracellular
M_nh4_c	Ammonium	Cytoplasm
M_o2_b	O2	Extracellular
M_o2_c	O2	Cytoplasm
M_oaa_c	Oxaloacetate	Cytoplasm
M_pep_c	Phosphoenolpyruvate	Cytoplasm
M_pyr_c	Pyruvate	Cytoplasm
M_q8_c	Ubiquinone-8	Cytoplasm
M_q8h2_c	Ubiquinol-8	Cytoplasm
M_r5p_c	alpha-D-Ribose-5-phosphate	Cytoplasm
M_ru5p_D_c	D-Ribulose-5-phosphate	Cytoplasm
M_s7p_c	Sedoheptulose-7-phosphate	Cytoplasm
M_succ_b	Succinate	Extracellular
M_succ_c	Succinate	Cytoplasm
M_succoa_c	Succinyl-CoA	Cytoplasm
M_xu5p_D_c	D-Xylulose-5-phosphate	Cytoplasm
M_xylo_b	D-Xylose	Extracellular
M_xylo_c	D-Xylose	Cytoplasm
M_xylu_c	D-Xylulose	Cytoplasm

Table S2. Reaction weights. Each reaction was weighted according to the number of independent enzymes or enzyme complexes catalyzing a reaction in parallel. If for a reaction the gene-enzyme-reaction mapping was missing we assigned a weight of 999. Thus making it very hard to delete such a reaction. On the other hand, uptake reactions were associated with weights of 0.1, as these are easy to “delete” by simply removing the substrate from the growth medium.

Reaction	Weight	Reaction	Weight	Reaction	Weight
R_ANA1	1	R_GG2r	1	R_TCA1	2
R_ANA2	2	R_GG3	2	R_TCA10	1
R_ANA3	1	R_GG4	2	R_TCA2r	1
R_ARA1	3	R_GG5r	2	R_TCA3r	1
R_BIO	999	R_GG6r	1	R_TCA4	1
R_EDP1	1	R_GG7r	1	R_TCA5	2
R_EDP2	1	R_GG8r	1	R_TCA6r	1
R_FC1r	1	R_GG9r	3	R_TCA7	1
R_FC2	1	R_GLB1	1	R_TCA8r	1
R_FEM1	2	R_GLB2	2	R_TCA9r	1
R_FEM2	1	R_MAN1	0.1	R_TRA1	999
R_FEM3	1	R_MAN2	1	R_TRA10	0.1
R_FEM4	2	R_OPM1	3	R_TRA11	0.1
R_FEM5	1	R_OPM2	2	R_TRA2	999
R_FEM6	3	R_OPM3	999	R_TRA3	0.1
R_FEM7	1	R_OPM4r	1	R_TRA4	999
R_FEM8	1	R_PPP1	1	R_TRA5	999
R_FEM9	1	R_PPP2	1	R_TRA6	999
R_GAL1	2	R_PPP3	1	R_TRA7	999
R_GG1	0.1	R_PPP4r	1	R_TRA8	0.1
R_GG10r	1	R_PPP5r	2	R_TRA9	0.1
R_GG11	2	R_PPP6r	1	R_XYL1	1
R_GG12	1	R_PPP7r	1	R_XYL2	1
R_GG13	2	R_PPP8r	1		

Table S3. Number of MCS as function of number of deletions for the complete Trinh-model (Trinh *et al.*, 2008) designed for optimal production of ethanol from glucose. In total we found 55,488 MCS.

Deletions	MCS	Experimentally feasible MCS
11	1,440	0
12	8,928	768
13	20,928	5,376
14	18,816	6,912
15	5,376	2,304
Total	55,488	15,360

Robustness of optimal solutions against variations in the weight vector

In the case of anaerobic growth we analyzed the impact of the weight function by repeating our calculation for various weight vectors . We used the weights given in the supplementary material, Table S2 as starting values. The components of new weight vectors, $\boldsymbol{w}^{(l)}$, were generated by applying the function, $f^{(l)}(x) = x^{0.95^l}$, $l \in \{0, 1, \ldots\}$, element-wise to the starting weight vector, $\boldsymbol{w}^{(0)}$. The choice of $f^{(l)}(x)$ is motivated by the property $\lim_{l\to\infty} \boldsymbol{w}^{(l)} = \mathbf{1}$, which (re-)covers the situation without weights. In $f^{(l)}(x)$ we used an exponent of 0.95 to guarantee that in the first step all weights not equal to one change at least by 10% of their initial value. Numerically we round $f^{(l)}(x)$ to two decimal places.

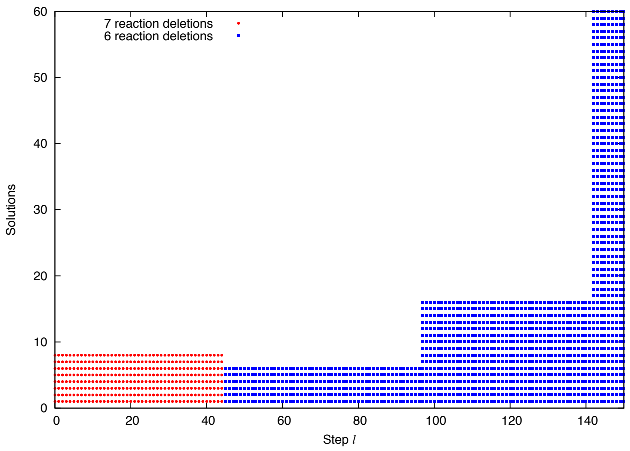

Figure S1. Number of alternate optimal solutions at the maximum of $[\boldsymbol{w}^{(l)}]^{\mathrm{T}}\boldsymbol{x}$ as function of l. Each symbol marks a single solution. Circles and squares represent minimal cut sets which require seven and six reaction deletions, respectively.

In supplementary material, Figure S1 we show the number of alternate optimal solutions at the maximum of $[\boldsymbol{w}^{(l)}]^{\mathrm{T}}\boldsymbol{x}$ as function of l. At $l = 0$ weights according to the supplementary material, Table S2 are used. As detailed above, we find eight alternate MCS. Each MCS requires 7 reaction deletions. Up to $l = 44$ we find the same eight solutions. At $l = 45$ initial weights of 0.10, 1.00, 2.00, 3.00, and 999.00 become 0.80, 1.00, 1.07, 1.12, and 1.99, respectively. At this time knocking out reactions weighted with 1.99 contribute less to the overall objective function than any pair of reactions weighted with 1.00, 1.07 or 1.12. In our model reactions with a weight of 999.00 (at $l = 0$; 1.99 at $l = 45$) are typically non-enzymatic transport reactions. Thus at $l = 45$ it is – for the first time – “better” to knockout a single transport reaction than to delete two enzymatic, non-uptake reactions. This is why the total number of required knockouts drops to six. We found six alternate solutions. If $l \geq 97$, then $f^{(l)}(2) = 1.00$; thus reactions originally weighted with a factor of two are know as cheap to delete as reactions originally weighted with one. This is why the number of alternate solutions rises at $l = 97$ form six to 16. The same argument applies at $l = 142$, where $f^{(142)}(999) = 1.00$. At this time the initial weight vector is converted in the $\mathbf{1}$-vector and we recover the same result as without weights. We find 60 alternate solutions, in agreement with Hädicke and Klamt (2011). Note that in all these cases, uptake reactions are not part of any MCS and therefore do not show up in this analysis.

REFERENCES

Hädicke, O. and Klamt, S. (2011). Computing complex metabolic intervention strategies using constrained minimal cut sets. *Metabolic Engineering*, **13**(2), 204–213.

Trinh, C. T., Unrean, P., and Srienc, F. (2008). Minimal escherichia coli cell for the most efficient production of ethanol from hexoses and pentoses. *Applied and Environmental Microbiology*, **74**(12), 3634 –3643.
